# Insulin Exacerbates Inflammation in Fibroblast-Like Synoviocytes

**DOI:** 10.1007/s10753-020-01178-0

**Published:** 2020-01-24

**Authors:** Li Qiao, Yi Li, Shui Sun

**Affiliations:** Department of Joint Surgery, Shandong Provincial Hospital Affiliated to Shandong University, Jinan, 250021 China

**Keywords:** osteoarthritis, insulin, fibroblast-like synoviocytes, inflammation, autophagy

## Abstract

Osteoarthritis (OA) is considered the most frequent degenerative disease and is characterized by cartilage degradation and synovial inflammation. Fibroblast-like synoviocytes (FLSs) are vital to synovial inflammation in OA. Type 2 diabetes mellitus (T2DM) is characterized by insulin resistance and hyperinsulinemia (HINS) and has been demonstrated to be an independent risk factor for OA. Autophagy is involved in the processes of various inflammatory diseases, and autophagy inhibition can stimulate OA development. Thus, we aimed to investigate the role of insulin in the inflammatory phenotype and autophagy of FLSs in OA. The data showed that cell viability and proinflammatory cytokine production in FLSs were both increased after insulin stimulation. We also found that high insulin could promote macrophage infiltration and chemokine production but inhibited autophagy in FLSs. To further explore the potential mechanisms, the effects of insulin on PI3K/Akt/mTOR and NF-ĸB signaling activation were evaluated. The results indicated that insulin activated PI3K/Akt/mTOR/NF-ĸB signaling, and the above-mentioned inflammatory responses, including autophagy inhibition, were notably attenuated by specific signaling inhibitors in the presence of high insulin. Moreover, the data showed that a positive feedback loop existed between proinflammatory cytokines (*e.g.*, IL-1β, IL-6, and TNF-α) and PI3K/mTOR/Akt/NF-ĸB signaling in FLSs, and insulin enhanced this feedback loop to accelerate OA progression. Our study suggests that insulin may be a novel therapeutic strategy for OA prevention and treatment in the future.

## INTRODUCTION

Osteoarthritis (OA) is an underlying cause of disability and the most frequently occurring degenerative joint disease [1]. Synovial inflammation, including cytokine secretion and inflammatory cell infiltration (primarily macrophages), is one of the characteristic pathological hallmarks of OA [2–5]. There has been increasing evidence from ongoing research that fibroblast-like synoviocytes (FLSs) are vital to the pathogenesis of OA. The synovial membrane, which is important for maintaining the intra-articular environment, can generate synovial fluid to maintain joint activity. The synovial membrane is also one of the major sources of intra-articular inflammation and can sustain and even worsen the prognosis of OA by synthesizing proinflammatory cytokines, such as interleukins (ILs) and tumor necrosis factors (TNFs) [6–8]. IL­-1β, IL-­6, and TNF-α have been reported as the major proinflammatory cytokines participating in the pathophysiology of OA. The synergistic effects of these three cytokines can facilitate OA [2, 3, 9, 10]. Along with FLSs, macrophages also act as crucial components in synovial tissues that participate in OA pathogenesis. Indeed, the degree of macrophage infiltration is a characteristic of OA and is positively correlated with disease severity [4, 5]. Thus, synovial inflammation has attracted substantial attention because of its potential as a therapeutic target and its involvement in OA pathogenesis. It is known that the occurrence and development of OA are closely associated with numerous cell signaling pathways. The classical nuclear factor-kappa B (NF-ĸB) signaling pathway has been reported to be crucial for inducing the expression of various proinflammatory cytokines (*e.g.*, IL-­1β, IL-­6, and TNF-α) and is considered a potential target in OA progression. Interestingly, these downstream cytokines (*e.g.*, IL­-1β, IL-­6, and TNF-α) are also important inducers of NF-ĸB signaling. As a result, this process could form a positive feedback loop to accelerate OA progression [2, 11, 12]. Moreover, proinflammatory cytokines induced by the NF-ĸB signaling pathway (*e.g.*, IL­-1, IL-­6, and TNF-α) could lead to matrix metalloproteinase (MMP) production, causing articular cartilage breakdown and bone resorption in OA progression. Among the MMP family members, MMP-13 and MMP-9 have been reported to be vital for OA progression [13, 14]. Thus, the potential role of the NF-ĸB signaling pathway and the targets of inflammatory responses in FLSs were explored in this study.

Recent studies have suggested that metabolic syndrome is closely associated with OA. Indeed, clinical studies have verified that type 2 diabetes mellitus (T2DM), an independent risk factor for OA, is closely associated with the pathological mechanism of OA [15, 16]. Abnormal insulin secretion is a critical characteristic of the early stages of T2DM, and T2DM manifests primarily as hyperinsulinemia (HINS) and insulin resistance. Increased insulin levels can lead to a wide range of effects on metabolism, including increased OA-related proinflammatory cytokine (such as ILs, TNF-α, MMP-13) levels [16–19]. Furthermore, by preventing chondrocyte maturation and inducing cartilage degeneration, insulin can aggravate the pathological variations of OA [19, 20].

Autophagy, a critical process for maintaining homeostasis, can significantly inhibit inflammation and inflammatory disease progression (*e.g.*, OA) [21–23]. There has been increasing evidence that autophagy deregulation, especially autophagy inhibition, is closely associated with the pathogenesis of OA [23–25]. In the process of autophagy, microtubule-associated protein 1 light chain 3 I (LC3I) is converted to microtubule-associated protein 1 light chain 3 II (LC3II) in autophagosomes. Thus, the expression level of LC3II, an autophagosome-specific marker protein, reflects the number of autophagosomes and thus the autophagy activity [26, 27]. Although the signaling regulators involved in autophagy are sophisticated, the PI3K/Akt/mTOR pathway has been well accepted as a basic intracellular signaling pathway, and it can inhibit autophagy when activated in OA progression [28, 29]. Moreover, the PI3K/Akt/mTOR pathway has long been considered as the key signaling pathways in cell growth and survival, and the activating factors of this pathway include insulin [30, 31]. In addition, a previous study successfully showed that insulin can exacerbate cartilage degeneration and facilitate OA progression by inhibiting autophagy through the mTOR pathway [20]. Based on the above-mentioned analysis, this study aimed to analyze the inhibitory effect of the PI3K/AKT/mTOR signaling pathway on autophagy and inflammatory responses in FLSs.

The independent effects of high insulin on the biological behavior of FLSs (*e.g.*, proliferation, autophagy, chemotactic effect on macrophages, and inflammatory activity) have never been studied in the literature. This study aimed to explore whether a potential link may exist between T2DM and OA to gain insight into the pathogenesis of OA and introduce new ideas for the clinical management of this disease.

## MATERIALS AND METHODS

### Synovial Tissue Collection

Synovial tissues were collected from patients with OA during knee joint replacement surgery (*n* = 39; 14 males and 25 females, aged 52 to 79 years old, mean age 63 years old). All patients satisfied the American College of Rheumatology (ACR) diagnostic criteria for OA. Informed consent was obtained from all individual participants included in the study. All experimental procedures for the use of human samples in this study were approved by the Ethics Committee of Shandong Provincial Hospital Affiliated to Shandong University (NO. 2017-53).

### Cell Culture

OA synovial tissues were macerated, chopped, and then incubated with type II collagenase (1 mg/ml, Sigma) in Dulbecco’s modified Eagle’s medium (DMEM, HyClone, Thermo Scientific) for 6 h at 37 °C and 5% CO_2_ (Thermo Scientific). The tissues were treated with 0.25% trypsin (Solabio) diluted in a phosphate-buffered saline (PBS) solution at a volume equivalent to that of DMEM. The cells were filtered; incubated overnight in DMEM supplemented with 10% fetal bovine serum (FBS, HyClone, Thermo Scientific), penicillin (100 IU/ml), and streptomycin (100 μg/ml, Gibco); and passaged three times. FLSs at passages 4–6 were used for our study.

### Stimulation Assays

FLSs were plated on 6-well plates ( 3-5× 10^5^ cells/well) or 24-well plates (8× 10^4^ cells/well) in high glucose DMEM containing 10% or 2% FBS. Insulin (0, 100, 200, or 500 nM; Sigma-Aldrich, St. Louis, MO, USA; I0516) or inflammatory stimulators, including IL-1β, IL-6, and TNF-α (0, 0.01, 0.1, 1, 10, or 50 ng/ml), were added and then incubated for the indicated times. PBS was used as a control. PDTC (an NF-ĸB inhibitor; MCE, USA), LY294002 (a PI3K/Akt inhibitor; MCE, USA), and rapamycin (an mTOR inhibitor; MCE, USA) were used to block the signaling pathways, and an equal amount of inhibitor solvent (0.1% DMSO, 99.9% corn oil) was used as a control.

### Cell Counting Kit-8

Cell suspensions were seeded on a 96-well plate at 1000 cells/well. Next, the cells were shaken, incubated for 4 to 6 h in a 37 °C incubator until they adhered, and incubated with insulin (0, 100, 200, or 500 nM) and with or without each of the inhibitors (LY294002 or rapamycin [10 μM]). The control group received the same amount of solvent. After 24 h or 48 h, 100 μl of CCK-8 reagent was added to each well, shaken, and subsequently incubated for 2 h. Afterward, the absorbance at 450 nm was measured with a microplate reader.

### Transwell

According to the source of the supernatant in the lower chamber, the experiments were divided into four groups: the control (N) group, the insulin (ISN)-mediated group (INS group), the fibroblast-like synoviocyte (FLS)-mediated group (FLS group), and the INS- and FLS-mediated group (INS+FLS group). When the FLSs in the 24-well plate reached 80% confluence, the DMEM was replaced with fresh DMEM or with DMEM containing a high INS concentration (500 nM). After 24 h, 1000 μl of the supernatant from each FLS-plated well under different stimulation conditions (DMEM with or without 500 nM INS) was aspirated and mixed well, and then 600 μl of it was added to a lower Transwell chamber (FLS and INS+FLS groups). The supernatant for the control group was 600 μl of DMEM, and the supernatant for the INS group was 600 μl of DMEM with 500 nM INS; these supernatants were placed in other lower Transwell chambers (N and INS groups) to start the experiments. When FLSs in a 24-well plate reached 80% confluence, insulin (500 nM) was added for 24 h. Then, 600 μl of the supernatant was aspirated and added to the lower Transwell chamber. After macrophage digestion, a cell suspension was prepared with DMEM/F12 medium, and 200 μl was added to the upper Transwell chamber. Next, the cells were incubated overnight, and the cells that did not migrate were wiped away with a cotton swab. Then, the cells in the lower chamber were stained with hematoxylin for 10 min at ambient temperature. The number of migrated cells was examined using a cell counter with microscopic imaging system.

### Reverse Transcription-Quantitative Polymerase Chain Reaction

FLSs were plated in 24-well plates and cultured in the presence of insulin (0, 100, 200, or 500 nM), the inflammatory stimulators IL-1β, IL-6, and TNF-α (0, 0.01, 0.1, 1, 10, or 50 ng/ml), or different signaling inhibitors (PDTC, LY294002, or rapamycin); then, the cells were analyzed 6, 12, or 24 h after treatment. Total RNA was extracted from FLSs using TRIzol reagent (Invitrogen) and reverse-transcribed using a ReverTra Ace qPCR RT kit (Toyobo, Japan) following the manufacturer’s protocol. Reverse transcription-quantitative polymerase chain reaction (RT-qPCR) was performed using a LightCycler 480 (Roche, Basel, Switzerland) according to the following protocol: denaturation at 95 °C for 10 min, 40 cycles of denaturation at 95 °C for 10 s, annealing at 60 °C for 1 min, and extension at 72 °C for 1 s. The primers for RT-qPCR were designed according to the consensus sequences by Oligo. GAPDH was used as an internal loading control. The forward and reverse primers are listed in Table 1, and all primers were created by BGI (Beijing, China). To measure the relative messenger RNA (mRNA) levels, the 2-ΔΔ cycle threshold (2-ΔΔCT) method was used.

**Table 1:** Primers used for real-time polymerase chain reaction

Primer name	Primer base sequence (5′ to 3′)
GAPDH	Forward:CACCATCTTCCAGGAGC Reverse:AGTGGACTCCACGACGTA
IL-1β	Forward:CTAAAGTATGGGCTGGACTG Reverse:AGCTTCAATGAAAGACCTCA
IL-6	Forward:ACTCACCTCTTCAGAACGAATTG Reverse:CCATCTTTGGAAGGTTCAGGTTG
TNFα	Forward:TGTCTACTGAACTTCGGGGT Reverse:TCACAGAGCAATGACTCCAA
MMP-9	Forward:TTGACAGCGACAAGAAGTGG Reverse:GCCATTCACGTCGTCCTTAT
MMP-13	Forward:CGCGTCATGCCAGCAAATTCCATT Reverse:TCCATGTGTCCCATTTGTGGTGTG
IL1R1	Forward:TTCCTGCTAAGGTGGAGGATTC Reverse:GCTCATTCTCCACAAATTTTGC
IL1R3	Forward:CTCAGAACGCTGCGATGACT Reverse:CGGTCCTGCCTAGTCCAATAC
IL6R	Forward:TCTGGAAACTATTCATGCTACCG Reverse:ACTCACAAACAACATTGCTGAGG
GP130	Forward:TCTGGAAACTATTCATGCTACCG Reverse:ACTCACAAACAACATTGCTGAGG
TNFR1	Forward:AGGAGAAACAGAACACCGTGTG Reverse:CCCTTAACATTCTCAATCTGGG
TNFR2	Forward:GACTTCATCCACGGATATTTGC Reverse:ACACTGGCTGGGGTAAGTGTAC

*IL* interleukin, *TNF* tumor necrosis factor, *MMP* matrix metalloproteinase, *IL1R* interleukin-1 receptor, *IL6R* interleukin-6 receptor, *GP* glycoprotein, *TNFR* tumor necrosis factor receptor

### Enzyme-Linked Immunosorbent Assay

Cells were cultured and then stimulated as described above, and the supernatants were collected at 6 h, 12 h, or 24 h. The release of proinflammatory cytokines (IL-1β, IL-6, and TNF-α), matrix metalloproteinases (MMP-9 and MMP-13), and chemokines (CXCL12, CCL2/MCP-1, and CCL5/RANTES) was analyzed using enzyme-linked immunosorbent assay (ELISA) kits (Multi Sciences, Hang Zhou, China) following the manufacturer’s instructions.

### Western Blotting

Whole cell lysates were separated by SDS-polyacrylamide gel electrophoresis (SDS-PAGE) and then transferred onto a polyvinylidene difluoride membrane (Merck Millipore, Darmstadt, Germany). Western blotting was performed using anti-LC3I (1:1000, Abcam, USA), anti-LC3II (1:1000, Abcam, USA), anti-PI3K (1:1000, CST), anti-phospho-PI3K (1:500, CST), anti-Akt (1:3000, CST), anti-phospho-Akt (1:1000, CST), anti-mTOR (1:3000, CST), anti-phospho-mTOR (1:1000, CST), anti-phospho-p50 NF-ĸB (1:1000, CST), p50 NF-ĸB (1:2000, CST), anti-phospho-p65 NF-ĸB (1:1000, CST), and anti-p65 NF-ĸB (1:2000, CST) antibodies. GAPDH (1:10000, Abcam, USA) was used as a loading control for proteins. The band intensities were analyzed using an ECL Plus detection system (Thermo Scientific, Pittsburgh, PA, USA).

### Immunofluorescence

FLSs were grown in 6-well plates with high insulin stimulation (500 nM) for 24 h. Preconditioned cells were washed slowly three times with PBS for 5 min each, fixed with 4% paraformaldehyde for 30 min, washed three times with PBS (5 min each), and then treated with 5% bovine serum albumin (BSA) for 1 h. The cells were then incubated with anti-p50 (1:100 dilution) and anti-p65 (1:150 dilution) antibodies at 4 °C overnight. After the cells were washed slowly three times with PBS for 5 min each, FITC- and TRITC-conjugated secondary antibodies were used to visualize the proteins under a fluorescence microscope (Olympus, Tokyo, Japan). Nuclei were counterstained with 4′,6′-diamidino-2-phenylindole (DAPI).

### Statistical Analysis

Statistical analysis was conducted using the GraphPad Prism 5 software package (La Jolla, CA, USA). A *t* test was used to assess significant differences between two groups. The results of three different experiments are expressed as the mean ± SEM. Differences in the results with *P* < 0.05 were considered statistically significant.

## RESULTS

### FLSs Are Sensitive to Insulin

**Fig. 1:**
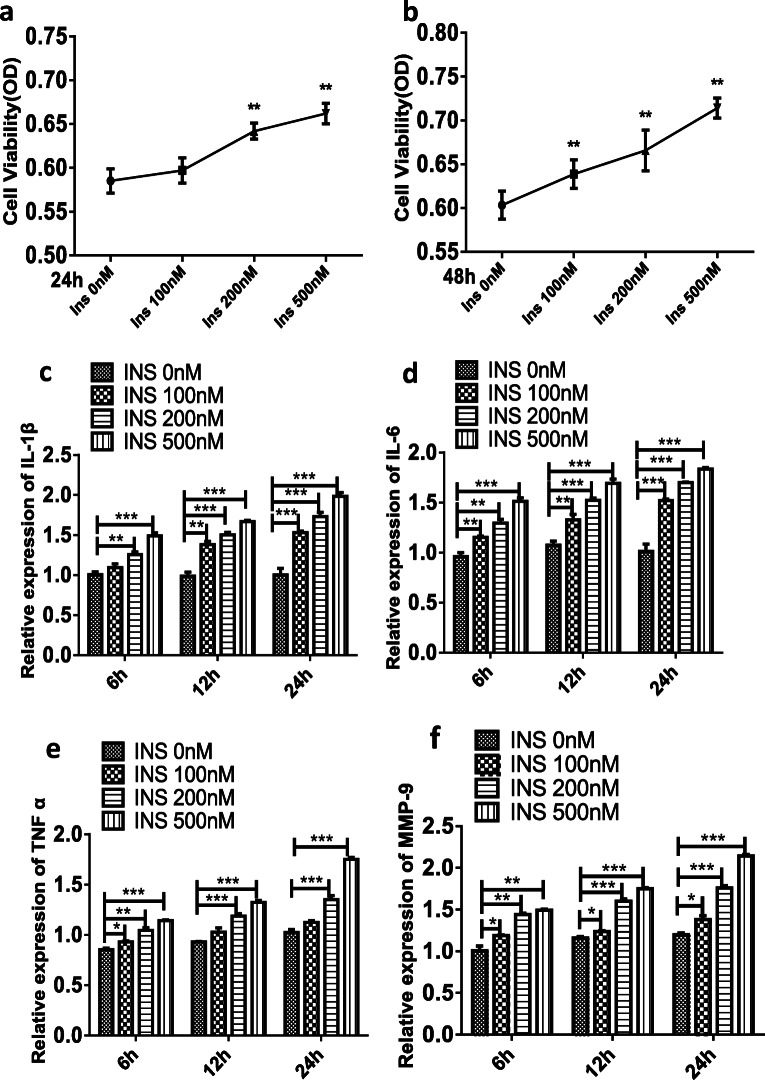

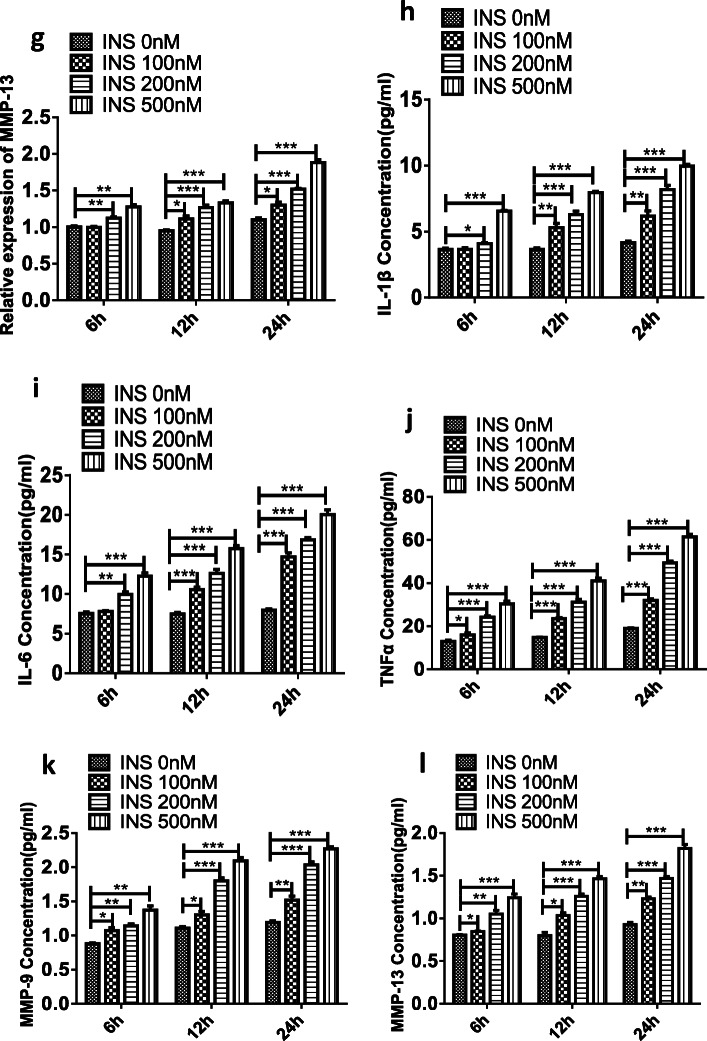
Cell viability and proinflammatory cytokines were both increased in response to high insulin in FLSs. **a**, **b** CCK-8 technology was used to assess cell viability of FLSs following insulin treatment (0, 100, 200, 500 nM) for 24 or 48 h; a representative of three independent experiments is shown. The figure of optical density (OD) detected by CCK-8 was used to reflect the degree of cell viability. FLSs were treated with insulin (0, 100, 200, 500 nM) for 6, 12, or 24 h. **c**–**g** The relative expression of IL-1β, IL-6, TNF-α, MMP-9, and MMP-13 were measured by RT-qPCR. **h**–**l** The secretion of IL-1β, IL-6, TNF-α, MMP-9, and MMP-13 were measured by ELISA. All the results are expressed as the mean ± SEM of three experiments conducted in triplicate separately. *P < 0.05, **P < 0.01, and ***P < 0.001 *vs.* control group.

A previous study suggested that FLSs are vital for osteoarthritis pathogenesis and that synoviocyte hyperplasia and abnormal synoviocyte proinflammatory cytokine production were both important pathological hallmarks of OA [2–5]. CCK-8 assays were applied to assess the effects of insulin (0, 100, 200, or 500 nM) on the viability of FLS. In response to insulin (200 or 500 nM), cell viability was significantly enhanced at 24 h (Fig. 1a) and 48 h (Fig. 1b). Next, the regulatory effects of insulin on proinflammatory cytokines and MMP mechanisms in FLSs were investigated. To determine whether insulin can directly regulate proinflammatory cytokine expression, FLSs were treated with incremental concentrations of insulin (0, 100, 200, or 500 nM). The qRT-PCR results (Fig. 1c–g) showed that IL-1β, IL-6, TNF-α, MMP-9, and MMP-13 expression levels were increased in a concentration-dependent manner compared with those of the control group (*P* < 0.05) at 6 h, 12 h, and 24 h after treatment with insulin (0, 100, 200, or 500 nM), and these levels were increased most significantly at 24 h. A similar increase in FLS secretion in response to insulin stimulation was observed by ELISA (Fig. 1h–l).

### Insulin Enhanced FLS-Mediated Chemotaxis in Macrophages

**Fig. 2:**
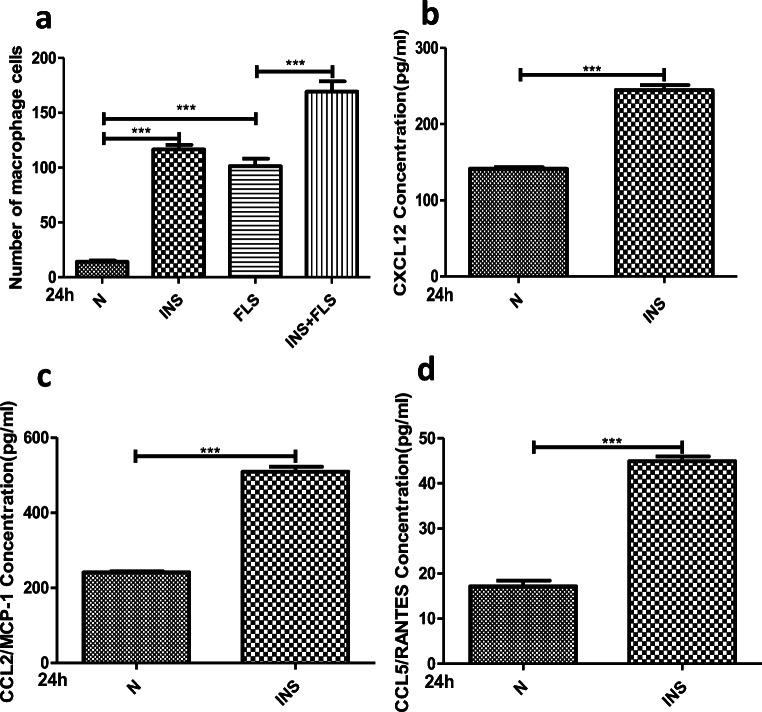
Effect of insulin on chemotaxis of FLSs to macrophages. FLSs received the treatment of high insulin (500 nM) for 24 h. **a** The chemotactic ability of FLSs was performed by Transwell assay and the average number of macrophage cells that invaded through the filter was quantified. Migration capacity of macrophage was measured by Transwell assay. **b**–**d** ELISA were performed to detect the secretion of CXCL12, CCL2/MCP-1, and CCL5/RANTES by high insulin (500 nM) after 24 h. All the results are expressed as the mean ± SEM of three experiments performed in triplicate separately. ***P < 0.001 *vs.* control group.

Because macrophage infiltration is a significant pathological feature of OA, macrophages can also contribute to OA [4, 5]. Chemokines are the major drivers of leukocyte adhesion and cell migration in inflammatory disease development [32, 33]. Among the chemokines, CXCL12, CCL2/MCP-1, and CCL5/RANTES can induce macrophage chemotaxis and are closely involved in OA development [34–36]. It is unclear whether insulin can regulate FLS-mediated macrophage infiltration and chemokine production. Transwell assays were employed to analyze the role of insulin in macrophage infiltration. The results suggest that the number of transmigrated macrophages was significantly increased at 24 h in the presence of FLSs treated with high insulin (500 nM). In addition, ELISA was used to detect CXCL12, CCL2/MCP-1, and CCL5/RANTES secretion by FLSs after 24 h. It was observed that insulin could independently attract macrophages in the absence of FLSs (Fig. 2a). Moreover, CXCL12, CCL2/MCP-1, and CCL5/RANTES secretion increased following insulin stimulation (500 nM, Fig. 2b–d).

### Insulin Activates the PI3K/mTOR/Akt and NF-ĸB Pathways and Mediates Autophagy Inhibition

**Fig. 3:**
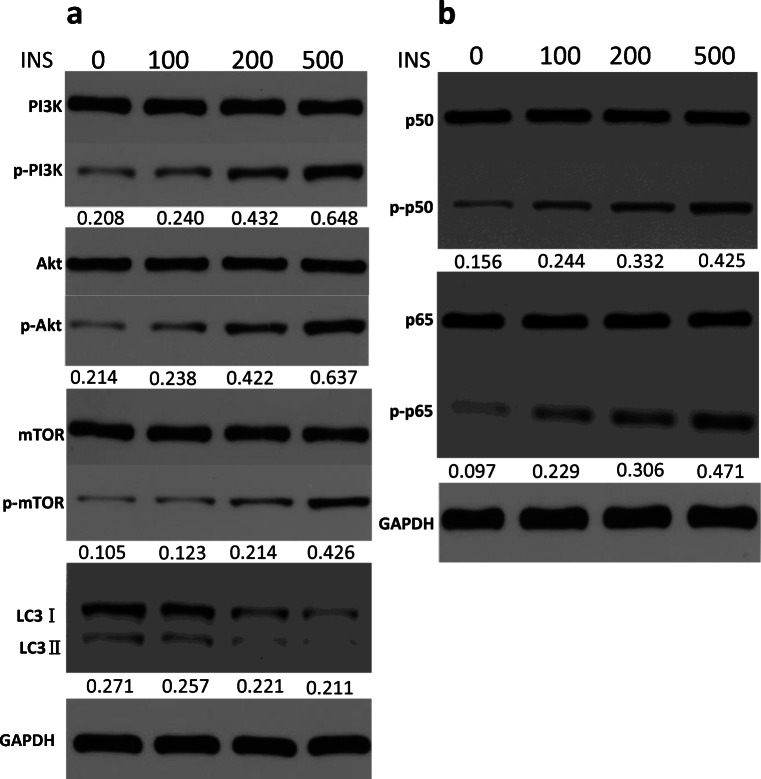

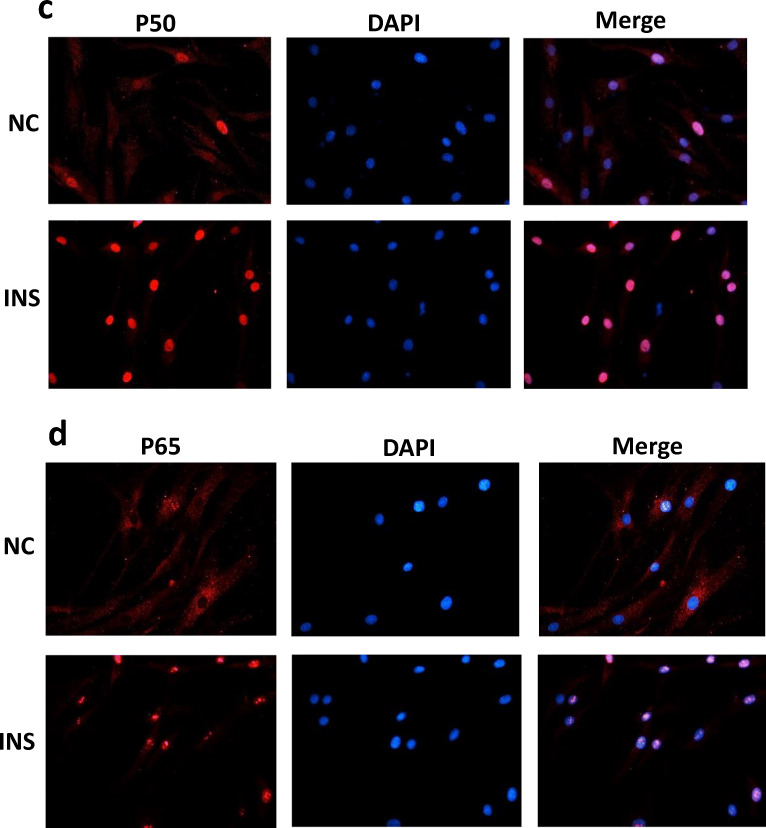
Activation of PI3K/Akt/Mtor/NF-ĸB pathway and autophagy deficiency by insulin. **a**, **b** Western blot analysis was used to examine the phosphorylation level of PI3K/Akt/mTOR and p50/p65 in FLSs treated with insulin (INS: 0, 100, 200, 500 nM) for 24 h. **c**, **d** Effects of insulin (INS: 500 nM) on p50 and p65 nuclear translocation in FLSs were observed using confocal fluorescence microscopy. **a** Western blot analysis was used to examine ratio of LC3 I/II in FLSs treated with insulin (Ins; 0, 100, 200, 500 nM) for 24 h. Results are expressed as the mean ± SEM of three experiments conducted in triplicate separately. All the results are expressed as the mean ± SEM of three experiments conducted in triplicate separately.

The above-mentioned data suggest that insulin could increase the inflammatory effect of FLSs. The PI3K/Akt/mTOR signaling pathway is involved in the pathogenesis of inflammation, including OA [28–31], and NF-ĸB is a known signaling pathway of OA inflammation [11, 12]. Thus, whether insulin can regulate PI3K/Akt/mTOR and NF-ĸB signaling pathway activation in FLSs was verified. Western blotting was used to evaluate the phosphorylation of PI3K/Akt/mTOR and p50/p65 (NF-ĸB subunits). According to the Western blotting results, the phosphorylation of PI3K/Akt/mTOR (Fig. 3a) and p50/p65 (Fig. 3b) was increased remarkably in FLSs treated with high insulin concentrations (100, 200, or 500 nM) for 24 h. These results verified PI3K/Akt/mTOR and NF-ĸB signaling pathway activation by high insulin conditions in FLSs. Immunofluorescence confirmed that a high insulin level (500 nM) could improve p50 and p65 (Fig. 3c, d) translocation into the nucleus in FLSs.

Autophagy is a critical cellular process that maintains homeostasis, and autophagy inhibition is closely associated with OA [21–25]. To evaluate how insulin regulates autophagy activation in FLSs, Western blotting was employed to detect the expression of LC3II, a major autophagy effector. LC3II expression and the LC3II/LC3I ratio were decreased with concomitantly increased PI3K/Akt/mTOR phosphorylation levels (Fig. 3a).

### Insulin Exacerbates Inflammatory Responses and Inhibits Autophagy in a Manner Dependent on the PI3K/mTOR/Akt and NF-ĸB Pathways

**Fig. 4:**
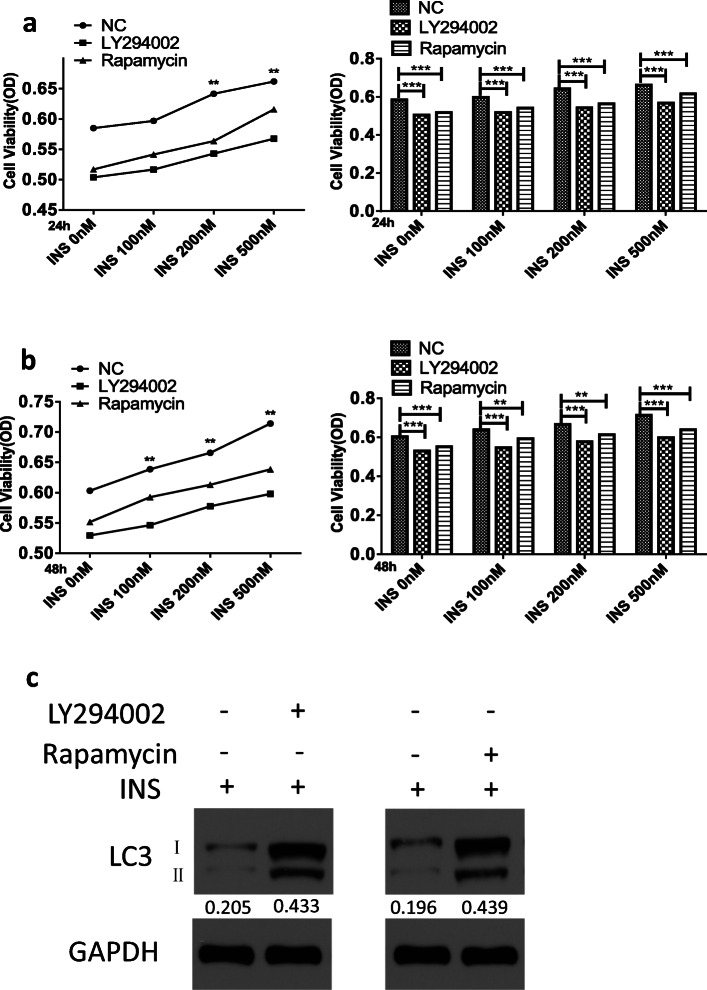

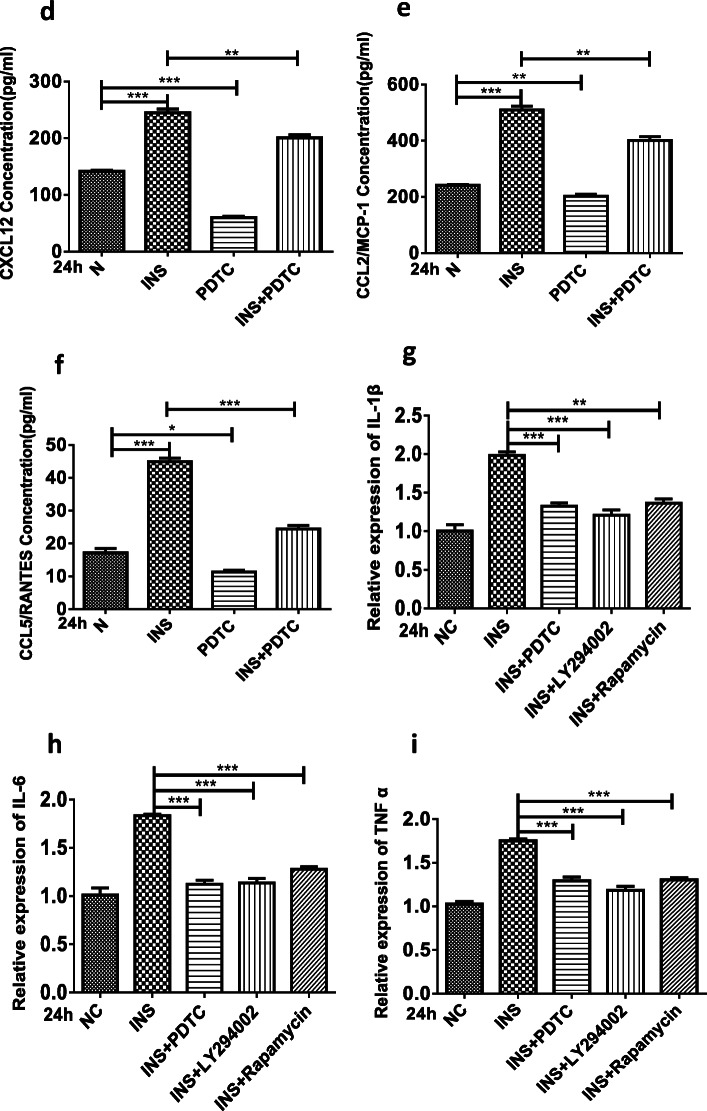

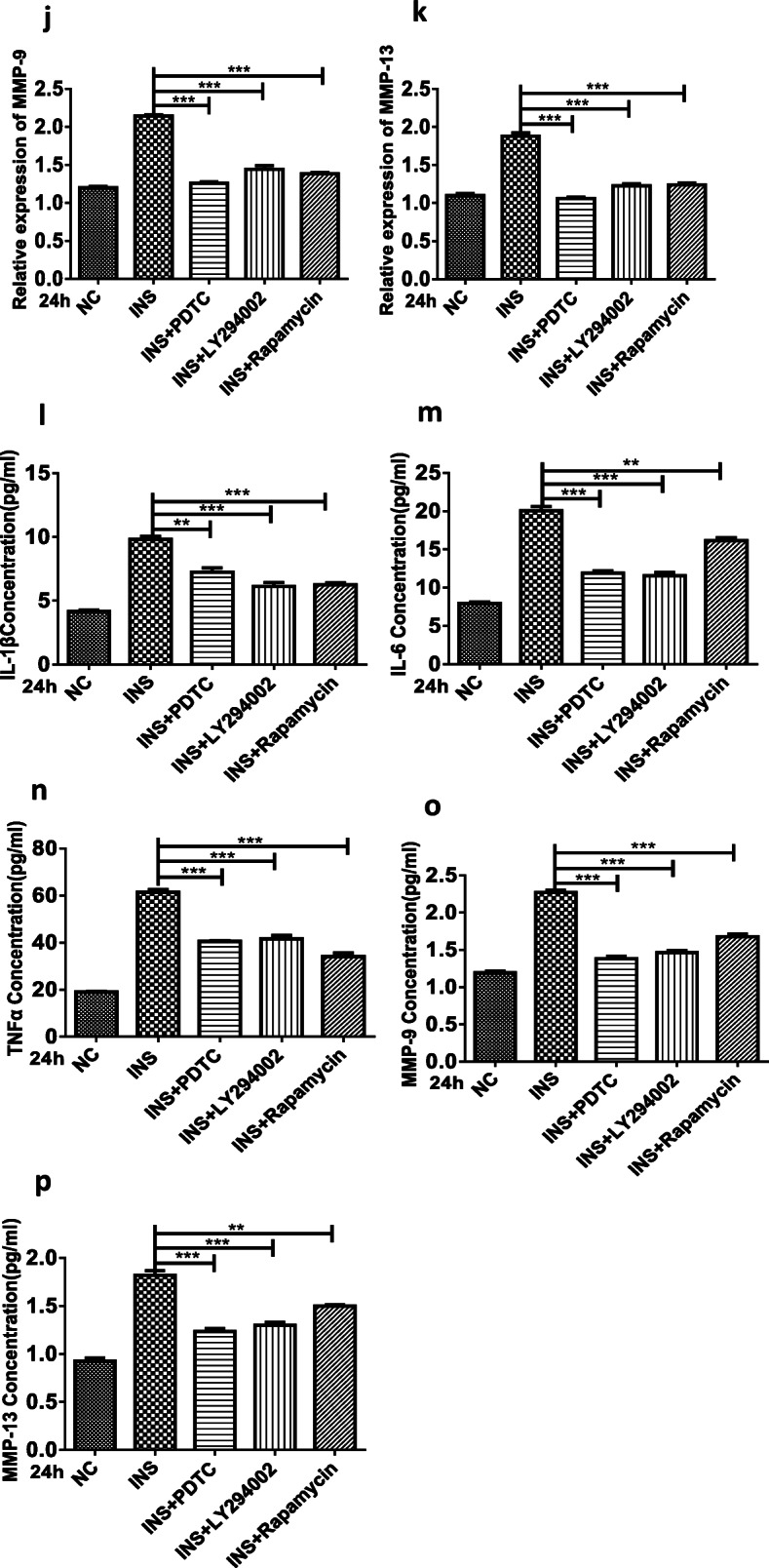
High insulin-mediated inflammatory responses are dependent on PI3K/Akt/mTOR and NF-ĸB pathway. **a**, **b** FLSs were treated with insulin (INS; 0, 100, 200, 500 nM) in the presence of inhibitor targeting PI3K/Akt (LY294002, 10 μM) and mTOR (Rapamycin, 10 μM) signaling. CCK-8 technology was used to assess cell viability of FLSs treated with a duration of 24 h or 48 h. **c** Various inhibitors targeting PI3K/Akt (LY294002, 10 μM) and mTOR (Rapamycin, 10 μM) were pre-treated for 6 h respectively to antagonize the effects of high insulin (500 nM) stimulation with a duration of 24 h. Ratio of LC3 I/II was determined by WB. **d**–**f** ELISA were performed to detect the secretion of CXCL12, CCL2/MCP-1, and CCL5/RANTES by high insulin (500 nM) in the presence of NF-ĸB inhibitor (PDTC, 10 μM) after 24 h. Various inhibitors targeting NF-ĸB (PDTC, 10 μM), PI3K/Akt (LY294002, 10 μM), and mTOR (Rapamycin, 10 μM) were pre-treated for 6 h to antagonize the effects of insulin stimulation with a duration of 24 h. **g**–**k** RT-qPCR was performed to ascertain the relative expression of IL-1β, IL-6, TNF-α, MMP-9, and MMP-13 in FLSs. **l**–**p** ELISA was performed to ascertain the secretion of IL-1β, IL-6, TNF-α, MMP-9, and MMP-13 in FLSs. Results are expressed as the mean ± SEM of three experiments conducted in triplicate separately. All the results are expressed as the mean ± SEM of three experiments conducted in triplicate separately. *P < 0.05. **P < 0.01. ***P < 0.001 *vs.* control group.

According to the above-mentioned data, insulin could activate the PI3K/Akt/mTOR and NF-ĸB signaling pathways in FLSs (Fig. 3a–c). In addition, the PI3K/Akt/mTOR pathway is a fundamental intracellular signaling pathway that is widely involved in autophagy regulation [28, 29]. To further verify the regulation of both signaling pathways by insulin, the effects of specific inhibitors on insulin-induced inflammatory response exacerbation and autophagy inhibition were also studied. The data revealed that inhibiting the PI3K/Akt/mTOR signaling pathways could eliminate the increased FLS viability following insulin treatment (0, 100, 200, or 500 nM) for 24 or 48 h (Fig. 4a, b). Furthermore, the decreases in LC3II expression and the LC3II/LC3I ratio caused by insulin (500 nM) could be restored by inhibitor treatment. The results demonstrated that the PI3K/Akt/mTOR pathway plays a role in autophagy regulation in FLSs (Fig. 4c).

Because NF-ĸB signaling could significcytokines (*e.g.*, IL-1β, IL-6, and TNF-α) directly and MMPs indirectly [11–14], we speculated that the overproduction of proinflammatory factors and chemokines following insulin treatment might result from NF-ĸB signaling pathway activation. To verify the mechanism of high insulin-induced proinflammatory factor and chemokine production, an inhibitor targeting the NF-ĸB (PDTC, 10 μM) signaling pathway was used. To evaluate the role of different pathways involved in OA progression, inhibitors targeting the PI3K/Akt/mTOR signaling pathway (LY294002, rapamycin) were employed. Subsequently, the inhibition of NF-ĸB signaling pathway could inhibit the CXCL12, CCL2/MCP-1, and CCL5/RANTES oversecretion induced by a high insulin concentration (500 nM) at 24 h (Fig. 4d–f). Similar results showing that inhibiting the PI3K/AKT/mTOR and NF-ĸB signaling pathways could reduce the overexpression (Fig. 4g–k) and secretion (Fig. 4l–p) of IL-1β, IL-6, TNF-α, MMP-9, and MMP-13 induced by a high insulin concentration (500 nM) at 24 h were also obtained. These experiments provide evidence that the proinflammatory effect of insulin on FLSs could be exerted through PI3K/mTOR/Akt and NF-ĸB pathway activation.

### Insulin Sensitizes FLSs to Inflammatory Factors by Upregulating Their Surface Receptors

**Fig. 5:**
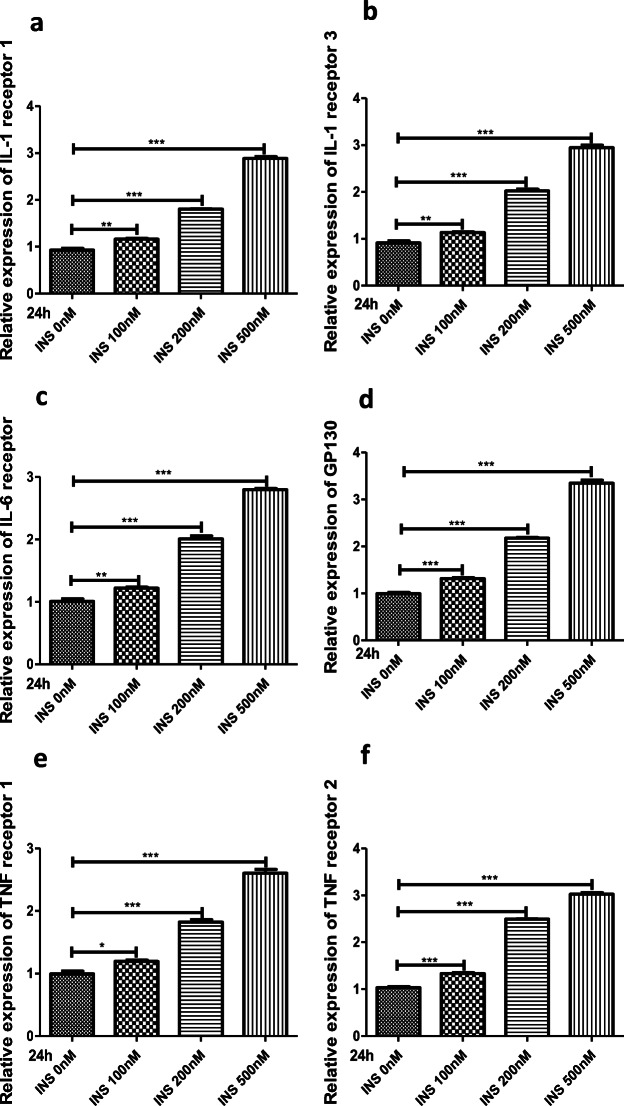

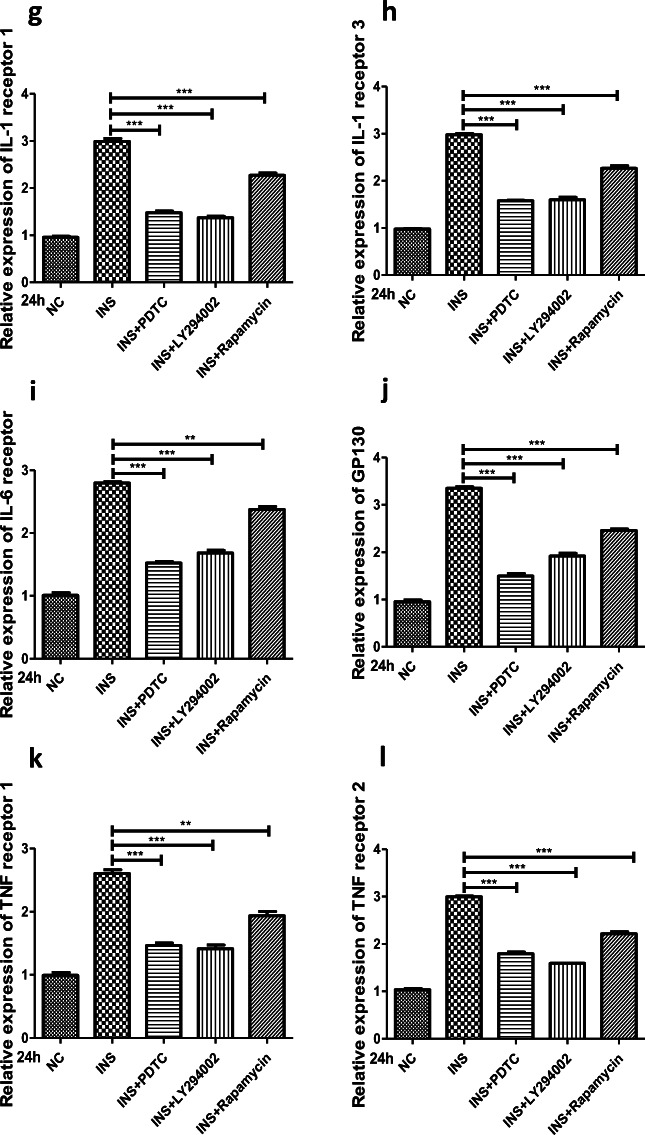
Insulin upregulated surface receptors of inflammatory factors through PI3K/Akt/mTOR and NF-ĸB pathway in FLSs. **a**–**f** FLSs were treated with insulin (0, 100, 200, 500 nM) for 6, 12, or 24 h. The expression and secretion of IL1R1, ILR3, IL6R, GP130, TNFR1, and TNFR2 were measured by qRT-PCR. **g**–**l** Various inhibitors targeting NF-ĸB (PDTC, 10 μM), PI3K/Akt (LY294002, 10 μM), and mTOR (Rapamycin, 10 μM) were pre-treated for 6 h to antagonize the effects of insulin stimulation with a duration of 24 h. To ascertain the expression and secretion of IL1R1, ILR3, IL6R, GP130, TNFR1, and TNFR2 in FLSs, qRT-PCR was performed. All the results are expressed as the mean ± SEM of three experiments conducted in triplicate separately. *P < 0.05. **P < 0.01. ***P < 0.001 *vs.* control group.

NF-ĸB contributes to the production of proinflammatory cytokines (*e.g.*, IL-1β, IL-6, and TNF-α), and in turn, the proinflammatory effects of IL-1β, IL-6, and TNF-α are mediated through the activation of several signaling pathways, including NF-ĸB signaling [2, 11, 12]. Thus, a feedback mechanism exists among inflammatory cytokines and signaling pathways, which can activate each other and have a close relationship. Based on the fact that insulin is a positive regulator of proinflammatory cytokine production and was found to upregulate the downstream targets of PI3K/mTOR/Akt/NF-ĸB signaling in FLSs, we hypothesized that insulin might be involved in the interaction between IL-1β/IL-6/TNF-α and PI3K/mTOR/Akt/NF-ĸB signaling. Cytokines act as important mediators of intercellular communication through specific cytokine receptors on the cell surface and initiate a series of specific biochemical reactions. The receptors responsible for the proinflammatory effects of IL-1β/IL-6/TNF-α-activated intracellular signaling pathways include interleukin-1 receptor 1 (IL-1R1), interleukin-1 receptor 3 (IL-1R3, interleukin-1 receptor accessory chain), interleukin-6 receptor (IL-6R), glycoprotein 130 (GP130, interleukin-6 coreceptor), tumor necrosis factor receptor 1 (TNFR1 or p55), and tumor necrosis factor receptor 2 (TNFR2 or p75) [37–39]. Thus, we measured the expression of these proinflammatory cytokine receptors in insulin-stimulated FLSs by qRT-PCR. The results showed that IL-1R1, IL-1R3, IL-6R, GP130, TNFR1, and TNFR2 expression levels were increased in a concentration-dependent manner compared with those of the control group (*P* < 0.05) at 24 h after treatment with insulin (0, 100, 200, or 500 nM); these receptors were upregulated most significantly at the 500 nM concentration (Fig. 5a–f). To explore the association of cytokine receptor upregulation with PI3K/mTOR/Akt/NF-ĸB signaling in FLSs, the effect of inhibitors targeting the PI3K/Akt (LY294002, 10 μM), mTOR (rapamycin, 10 μM), and NF-ĸB (PDTC, 10 μM) signaling pathways on insulin-induced cytokine receptor expression was studied. The data revealed that inhibiting the PI3K/Akt/mTOR and NF-ĸB signaling pathways could reduce the IL-1R1, IL-1R3, IL-6R, GP130, TNFR1, and TNFR2 overexpression induced by high insulin (500 nM) at 24 h (Fig. 5g–l). These experiments provide evidence that when FLSs are stimulated with insulin, the expression of proinflammatory cytokine (*e.g.*, IL­1-β, IL­6, and TNF-α) receptors depends on PI3K/Akt/mTOR and NF-ĸB signaling pathway activation.

### Insulin and Inflammatory Factors Synergistically Exacerbated the Inflammatory Phenotype of OA FLSs

**Fig. 6:**
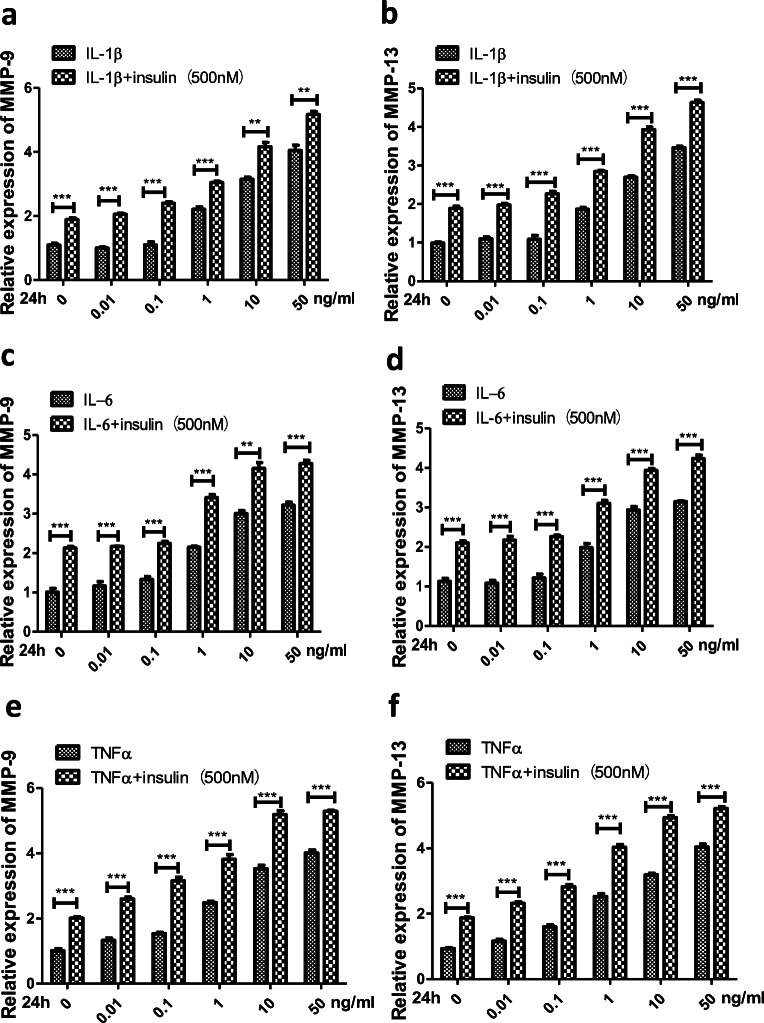

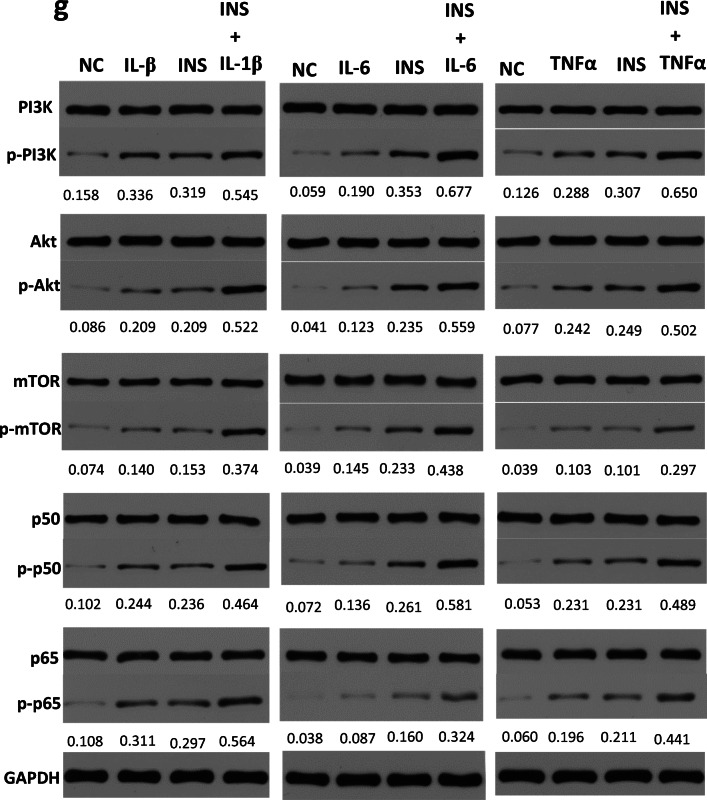
Effects of insulin on expression of MMPs and regulation of signaling pathways by inflammatory cytokines. **a**–**f** FLSs were treated for 24 h with IL-1β, IL-6, and TNF-α (0, 0.01, 0.1, 1, 10, 50 ng/ml) separately, or insulin (500 nM) was pre-treated for 12 h to affect the effects of inflammatory cytokines stimulation with a duration of 24 h. The expression of MMP-9 and MMP-13 were measured by qRT-PCR. **g** FLSs were treated for 24 h with IL-1β, IL-6, and TNF-α (50 ng/ml) separately, or insulin (INS:500 nM) was pre-treated for 12 h to affect the effects of inflammatory cytokines stimulation with a duration of 24 h. The phosphorylation level of PI3K/Akt/mTOR and p50/p65 were measured by Western blotting. All the results are expressed as the mean ± SEM of three experiments conducted in triplicate separately. **P < 0.01. ***P < 0.001 *vs.* control group.

In synovial joint pathology, a major hallmark in response to increased levels of IL-1β, IL-6 and TNF-α is the overexpression of MMPs, including MMP-9 and MMP-13, which contributes to cartilage degradation [13, 14]. To determine whether insulin can directly regulate cytokine-mediated inflammatory reactions, we chose MMP-9 and MMP-13 expression as a functional readout for the effect of insulin. FLSs were treated with incremental concentrations of IL-1β, IL-6 and TNF-α (0, 0.01, 0.1, 1, 10, or 50 ng/ml) or were cotreated with high insulin (500 nM) and all three cytokines separately for comparison. The qRT-PCR results showed that the expression of MMP-9 and MMP-13 increased (*P* < 0.05) after treatment with IL-1β, IL-6 and TNF-α (0, 0.01, 0.1, 1, 10, or 50 ng/ml) at 24 h; the MMPs were increased most significantly (*P* < 0.01) at the 50 ng/ml concentration, and the response was further increased by the presence of insulin (Fig. 6a–f).

Finally, we sought to investigate whether insulin could affect IL-1β, IL-6 and TNF-α-mediated signaling activation in FLSs, as suggested by the increase in cytokine-induced MMP expression. FLSs were treated with inflammatory cytokines (IL-1β, IL-6 and TNF-α, 50 ng/ml) and insulin (500 nM) separately or cotreated with the two stimuli simultaneously. Western blotting was used to test the phosphorylation of PI3K/Akt/mTOR and p50/p65 in different groups. After treatment, the PI3K/Akt/mTOR and p50/p65 phosphorylation levels in the three groups increased significantly and were even higher in the cotreatment group than in the other two groups (Fig. 6g). These experiments showed that insulin sensitized cellular signaling transduction activation by inflammatory cytokines. Based on the above-mentioned research, we suggest that insulin is involved in a positive feedback loop between IL-1β/IL-6/TNF-α and PI3K/mTOR/Akt/NF-ĸB signaling in FLSs.

## DISCUSSION

In this study, we first discovered that insulin can promote synovial inflammation in the progression of osteoarthritis, either alone or in conjunction with inflammatory factors. First, insulin can significantly promote the inflammatory phenotype of FLSs, increase cell viability and inflammatory cytokine production, promote chemokine production, and enhance macrophage chemotaxis. Second, insulin can activate the PI3K/mTOR/Akt/NF-ĸB signaling pathway and simultaneously inhibit autophagy in FLSs. Third, our data suggest that the insulin-induced inflammatory responses are significantly reduced by preblocking three signaling pathways with specific pathway inhibitors in FLSs. Finally, the data show that insulin can upregulate inflammatory cytokine receptor levels in FLSs, and PI3K/mTOR/Akt/NF-ĸB signaling inhibitors can reverse this process. More importantly, insulin has a sensitizing effect on inflammatory factor–mediated synovial inflammation (including MMP production and intracellular signaling pathway activation). Taken together, these data suggest that insulin may exacerbate synovial inflammatory lesions and thus contribute to the progression of OA.

Recent studies have demonstrated a close relationship between the pathogenesis of OA and metabolic syndrome based on insulin resistance and hyperinsulinemia [15, 16]. Nicola et al. found a positive correlation between T2DM, characterized by hyperinsulinemia, and the development of OA [16]. Previous study suggested that insulin could promote the production of various proinflammatory factors (such as ILs, TNF-α, MMP-13) associated with OA [16–19]. In addition, insulin can inhibit chondrocyte maturation and promote cartilage degradation *in vitro*, thus aggravating the pathological changes of OA [19, 20]. Synovial tissues, especially FLSs, have been shown to play a vital role in the development of OA [4–7]. However, until now, the effect of insulin on fibroblast synovial inflammation in OA was unclear. In this study, we found that insulin could exacerbate the inflammatory phenotype of OA FLSs. Thus, we hypothesized that insulin could promote OA progression through regulating the biological activity of various cell types.

Previous study showed that the PI3K/AKT and mTOR signaling pathways are implicated in the pathophysiological effects of insulin in OA [19, 40, 41]. NF-ĸB determines the expression of inflammatory factors and MMPs in the joints and is considered to play a very important role in the pathogenesis of OA [2, 11–14]. This study found that insulin can aggravate the inflammatory phenotype of fibroblast synovial cells by activating the PI3K/mTOR/Akt/NF-ĸB signaling pathways. Our results confirm previous research that the PI3K/mTOR/Akt/NF-ĸB pathway was involved in the proinflammatory effects of insulin towards FLSs. The production of inflammatory factors and activation of signaling pathways in OA FLSs were enhanced with the stimulation of insulin in combination with other proinflammatory factors, such as IL-1β, IL-6, and TNF-α, further supporting these findings. These results indicate that insulin can do more than promote synovial inflammatory lesions alone; it can also work synergistically with inflammatory factors, participate in and activate the positive feedback loop between inflammatory factors and signal pathways, and further amplify the synovial inflammation cascade.

Autophagy is an important cellular process that maintains homeostasis and is involved in the pathogenesis of many inflammatory diseases. Notably, autophagy inhibition is closely related to the pathogenesis of OA [25]. Studies by Ribeiro et al. [19] have shown that insulin can lead to proteoglycan component loss and inflammatory cytokine and MMP increases in chondrocytes, and these effects may be achieved by inhibiting chondrocyte autophagy. In addition, the clinical data of this study showed that autophagy decreased in the knee cartilage of diabetic patients compared with non-diabetic patients; namely, the expression of the autophagy-related protein LC3II decreased significantly. Notably, this study revealed that rapamycin, as an autophagy activator (also an mTOR signaling pathway inhibitor), can prevent insulin-mediated reductions in autophagy and cartilage degradation [19]. Our results showed that high insulin could also aggravate the inflammatory responses of FLSs by coordinating the autophagy process. These facts indicate that insulin was also related to autophagy in the pathogenesis of OA and provide a mechanistic basis for the role of insulin in OA.

Previous studies have suggested that the biological effects of insulin differs in a dose- and cell-specific manner [19, 20, 40–46]. In addition to the effects on FLSs shown in this study, chondrocytes were also found to be affected by insulin [19, 20, 40–43]. However, insulin has been reported to play either an inductive or inhibitory role in chondrocyte differentiation [20, 43] and consequently ameliorate or impair cartilage degeneration [19, 42] at different concentrations. Furthermore, at the molecular level, the influence in which insulin exerts differed greatly as its concentration is extremely high or low [40, 45, 46]. Rosa et al. [40] found that low and supraphysiological insulin levels exert different effects on aggrecan and proteoglycan synthesis in chondrocytes, which may be due to different receptors for insulin. Moreover, Mirdamadi [45] et al. found that high insulin (1 μM) reduced the FoxO transcriptional activity, while low insulin (0.1 μM) reduced the FoxO transcriptional activity of SZ95 sebocytes *in vitro*. Studies by Kubota et al. [46] showed that insulin selectively regulates specific downstream responses of the Akt pathway in a dose-dependent manner. Thus, we concluded that different mechanisms of insulin action were associated with different insulin concentrations, which may modulate the cellular response. Previous studies have shown that specific cellular responses could be achieved only after treatment with high concentrations of insulin [47–54]. Consistently, our data showed that insulin’s effects on FLSs, which were exerted in a concentration-dependent manner, were more obvious as the concentration of insulin is super physiological. Taken together with these studies, it is obvious that different effector cells involved in the complex pathophysiologic process of various diseases have different sensitivities to insulin [19, 40, 43, 47–52]. Our research object is FLSs involved in OA, and the different cell type selected is also the reason why the insulin concentration used in this study is different from that of other studies. As the insulin levels were significantly higher in the early stages of T2DM compared with those in non-diabetic individuals, our data may explain why patients with T2DM are more susceptible to OA than non-diabetic individuals. Therefore, although the insulin concentration chosen in our study is supraphysiological, our data still has reference value and clinical guiding significance.

In conclusion, our study demonstrated that insulin exacerbates the inflammatory phenotype involving PI3K/Akt/mTOR/NF-ĸB signaling of OA FLSs. Furthermore, autophagy was identified as another way for insulin to participate in the pathological progression of OA. Therefore, although the detailed regulatory mechanism needs to be further investigated, this study provides molecular evidence that insulin is closely related to OA by regulating the biological activity of FLSs, and blocking insulin-mediated pathway holds potential as a novel treatment strategy.

## Abbreviations

OA: Osteoarthritis
FLSs: Fibroblast-like synoviocytes
T2DM: Type 2 diabetes mellitus
HINS: Hyperinsulinemia
NF-ĸB: Nuclear factor–kappa B
IL: Interleukin
TNF: Tumor necrosis factor
MMP: Matrix metalloproteinase
LC3: Light chain 3

## References

[1] Global Burden of Disease Study 2013 Collaborators (2015). Global, regional, and national incidence, prevalence, and years lived with disability for 301 acute and chronic diseases and injuries in 188 countries, 1990–2013: a systematic analysis for the Global Burden Disease Study 2013. Lancet..

[2] Kapoor M, Martel-Pelletier J, Lajeunesse D, Pelletier JP, Fahmi H (2011). Role of proinflamatory cytokines in the pathophysiology of osteoarthritis. Nature Reviews Rheumatology.

[3] Wang T, He C (2018). Pro-inflammatory cytokines: the link between obesity and osteoarthritis. Cytokine & Growth Factor Reviews.

[4] Moradi B, Rosshirt N, Tripel E, Kirsch J, Barié A, Zeifang F, Gotterbarm T, Hagmann S (2015). Unicompartmental and bicompartmental knee osteoarthritis show different patterns of mononuclear cell infiltration and cytokine release in the affected joints. Clinical and Experimental Immunology.

[5] Klein-Wieringa IR, de Lange-Brokaar BJ, Yusuf E, Andersen SN, Kwekkeboom JC, Kroon HM, van Osch GJ, Zuurmond AM, Stojanovic-Susulic V, Nelissen RG, Toes RE, Kloppenburg M, Ioan-Facsinay A (2016). Inflammatory cells in patients with endstage knee osteoarthritis: a comparison between the synovium and the infrapatellar fat pad. Journal of Rheumatology.

[6] Wang X, Hunter DJ, Jin X, Ding C (2018). The importance of synovial inflammation in osteoarthritis: current evidence from imaging assessments and clinical trial. Osteoarthritis Cart.

[7] Bhattaram P, Chandrasekharan U (2017). The joint synovium: a critical determinant of articular cartilage fate in inflammatory joint diseases. Seminars in Cell & Developmental Biology.

[8] Scanzello CR, Goldring SR (2012). The role of synovitis in osteoarthritis pathogenesis. Bone.

[9] Pozgan U, Caglic D, Rozman B, Nagase H, Turk V, Turk B (2010). Expression and activity profiling of selected cysteine cathepsins and matrix metalloproteinases in synovial fluids from patients with rheumatoid arthritis and osteoarthritis. Biological Chemistry.

[10] Larsson S, Englund M, Struglics A, Lohmander LS (2015). Interleukin-6 and tumor necrosis factor alpha in synovial fluid are associated with progression of radiographic knee osteoarthritis in subjects with previous meniscectomy. Osteoarthritis and Cartilage.

[11] Rigoglou S, Papavassiliou AG (2013). The NF-kappaB signalling pathway in osteoarthritis. International Journal of Biochemistry & Cell Biology.

[12] Zheng W, Feng Z, Lou Y, Chen C, Zhang C (2017). Silibinin protects against osteoarthritis through inhibiting the inflammatory response and cartilage matrix degradation in vitro and in vivo. Oncotarget.

[13] Tokito A, Jougasaki M (2016). Matrix metalloproteinases in non-neoplastic disorders. International Journal of Molecular Sciences.

[14] Malemud CJ (2017). Matrix metalloproteinases and synovial joint pathology. Progress in Molecular Biology and Translational Science.

[15] Courties A, Sellam J, Berenbaum F (2017). Metabolic syndrome-associated osteoarthritis. Current Opinion in Rheumatology.

[16] Veronese N, Cooper C, Reginster JY, Hochberg M, Branco J, Bruere O, Chapurlat R, Al-Daghri N, Dennison E, Herrero-Beaumont G, Kaux JF, Maheu E, Rizzoli R, Roth R, Rovati LC, Uebelhart D, Vlaskovska M, Scheen A (2019). Type 2 diabetes mellitus and osteoarthritis. Seminars in Arthritis and Rheumatism.

[17] Lubberts E (2015). The IL-23-IL-17 axis in inflammatory arthritis. Nature Reviews Rheumatology.

[18] Berenbaum F (2011). Diabetes-induced osteoarthritis: from a new paradigm to a new phenotype. Annals of the Rheumatic Diseases.

[19] Ribeiro M, Lo’pez de Figueroa P, Blanco FJ, Mendes AF, Carame’s B (2016). Insulin decreases autophagy and leads to cartilage degradation. Osteoarthritis and Cartilage.

[20] Torres E, Andrade C, Fonseca E, Mello M, M. Duarte M. (2003). Insulin impairs the maturation of chondrocytes in vitro. Brazilian Journal of Medical and Biological Research.

[21] Feng Y, He D, Yao Z, Klionsky DJ (2014). The machinery of macroautophagy. Cell Research.

[22] Mizushima N, Komatsu M (2011). Autophagy: renovation of cells and tissues. Cell.

[23] Li YS, Zhang FJ, Zeng C, Luo W, Xiao WF, Gao SG, Lei GH (2016). Authophagy in osteoarthritis. Joint, Bone, Spine.

[24] Cheng NT, Meng H, Ma LF, Zhang L, Yu HM, Wang ZZ, Guo A (2017). Role of autophagy in the progression of osteoarthritis: the autophagy inhibitor, 3-methyladenine, aggravates the severity of experimental osteoarthritis. International Journal of Molecular Medicine.

[25] Caramés B, Olmer M, Kiosses WB, Lotz MK (2015). The relationship of autophagy defects to cartilage damage during joint aging in a mouse model. Arthritis & Rheumatology.

[26] Mizushima N, Yoshimori T, Levine B (2010). Methods in mammalian autophagy research. Cell.

[27] Caramés B, Hasegawa A, Taniguchi N, Miyaki S, Blanco FJ, Lotz M (2012). Autophagy activation by rapamycin reduces severity of experimental osteoarthritis. Annals of the Rheumatic Diseases.

[28] He W, Cheng Y (2018). Inhibition of miR-20 promotes proliferation and autophagy in articular chondrocytes by PI3K/AKT/mTOR signaling pathway. Biomedicine & Pharmacotherapy.

[29] Xue JF, Shi ZM, Zou J, Li XL (2017). Inhibition of PI3K/AKT/mTOR signaling pathway promotes autophagy of articular chondrocytes and attenuates inflammatory response in rats with osteoarthritis. Biomedicine & Pharmacotherapy.

[30] Wullschleger S, Loewith R, Hall MN (2006). TOR signaling in growth and metabolism. Cell.

[31] Engelman JA (2009). Targeting PI3K signaling in cancer: opportunities, challenges and limitations. Nature Reviews Cancer.

[32] Sallusto F, Baggiolini M (2008). Chemokines and leukocyte traffic. Nature Immunology.

[33] Bachelerie F, Ben-Baruch A, Burkhardt AM, Combadiere C, Faeber JM, Graham GJ, Horuk R, Sparre-Ulrich AH, Locati M, Luster AD, Mantovani A, Matsushima K, Murphy PM, Nibbs R, Nomiyama H, Power CA, Proudfoot AE, Rosenkilde MM, Rot A, Sozzani S, Thelen M, Yoshie O, Zlotnik A (2014). International union of basic and clinical pharmacology. LXXXIX. Update on the extended family of chemokine receptors and introducing a new nomenclature for atypical chemokine receptors. Pharmacological Reviews.

[34] Sánchez-Martín L, Estecha A, Samaniego R, Sánchez-Ramón S, Vega MÁ, Sánchez-Mateos P (2011). The chemokine CXCL12 regulates monocyte-macrophage differentiation and RUNX3 expression. Blood.

[35] Svensson S, Abrahamsson A, Rodriguez GV, Olsson AK, Jensen L, Cao Y, Dabrosin C (2015). CCL2 and CCL5 are novel therapeutic targets for estrogen-dependent breast cancer. Clinical Cancer Research.

[36] Scanzello CR (2017). Chemokines and inflammation in osteoarthritis: Insights from patients and animal models. Journal of Orthopaedic Research.

[37] Boraschi D, Italiani P, Weil S, Martin MU (2018). The family of the interleukin-1 receptors. Immunological Reviews.

[38] Baran P, Hansen S, Waetzig GH, Akbarzadeh M, Lamertz L, Huber HJ, Ahmadian MR, Moll JM, Scheller J (2018). The balance of interleukin (IL)-6, IL-6·soluble IL-6 receptor (sIL-6R), and IL-6·sIL-6R·sgp130 complexes allows simultaneous classic and trans-signaling. Journal of Biological Chemistry.

[39] Becker D, Deller T, Vlachos A (2015). Tumor necrosis factor (TNF)-receptor 1 and 2 mediate homeostatic synaptic plasticity of denervated mouse dentate granule cells. Scientific Reports.

[40] Rosa SC, Rufino AT, Judas F, Tenreiro C, Lopes MC, Mendes AF (2011). Expression and function of the insulin receptor in normal and osteoarthritic human chondrocytes: modulation of anabolic gene expression, glucose transport and GLUT-1 content by insulin. Osteoarthritis and Cartilage.

[41] Claassen H, Schlüter M, Schünke M, Kurz B (2006). Influence of 17beta-estradiol and insulin on type II collagen and protein synthesis of articular chondrocytes. Bone.

[42] Cai L, Okumu FW, Cleland JL, Beresini M, Hogue D, Lin Z, Filvaroff EH (2002). A slow release formulation of insulin as a treatment for osteoarthritis. Osteoarthritis and Cartilage.

[43] Phornphutkul C, Wu KY, Gruppuso PA (2006). The role of insulin in chondrogenesis. Molecular and Cellular Endocrinology.

[44] Rossetto R, Saraiva MVA, Bernuci MP, Silva GM, Brito IR, Alves AMCV, Magalhães-Padilha DM, Báo SN, Campello CC, Rodrigues APR, Figueiredo JR (2016). Impact of insulin concentration and mode of FSH addition on the in vitro survival and development of isolated bovine preantral follicles. Theriogenology.

[45] Kubota H, Noguchi R, Toyoshima Y, Ozaki Y, Uda S, Watanabe K, Ogawa W, Kuroda S (2012). Temporal coding of insulin action through multiplexing of the AKT pathway. Molecular Cell.

[46] Mirdamadi Y, Thielitz A, Wiede A, Goihl A, Papakonstantinou E, Hartig R, Zouboulis CC, Reinhold D, Simeoni L, Bommhardt U, Quist S, Gollnick H (2015). Insulin and insulin-like growth factor-1 can modulate the phosphoinositide-3-kinase/Akt/FoxO1 pathway in SZ95 sebocytes in vitro. Molecular and Cellular Endocrinology.

[47] Ho-Palma AC, Toro P, Rotondo F, Romero MDM, Alemany M, Remesar X, Fernández-López JA (2019). Insulin controls triacylglycerol synthesis through control of glycerol metabolism and despite increased lipogenesis. Nutrients.

[48] de Proença ARG, Pereira KD, Meneguello L, Tamborlin L, Luchessi AD (2019). Insulin action on protein synthesis and its association with eIF5A expression and hypusination. Molecular Biology Reports.

[49] Hodonu A, Escobar M, Beach L, Hunt J, Rose J (2019). Glycogen metabolism in mink uterine epithelial cells and its regulation by estradiol, progesterone and insulin. Theriogenology.

[50] Chen CD, Podvin S, Gillespie E, Leeman SE, Abraham CR (2007). Insulin stimulates the cleavage and release of the extracellular domain of Klotho by ADAM10 and ADAM17. Proceedings of the National Academy of Sciences of the United States of America.

[51] Montagnani M, Ravichandran LV, Chen H, Esposito DL, Quon MJ (2002). Insulin receptor substrate-1 and phosphoinositide-dependent kinase-1 are required for insulin-stimulated production of nitric oxide in endothelial cells. Molecular Endocrinology.

[52] Zeng G, Nystrom FH, Ravichandran LV, Cong LN, Kriby M, Mostowski H, Quon MJ (2000). Roles for insulin receptor, PI3-kinase, and Akt in insulin-signaling pathways related to production of nitric oxide in human vascular endothelial cells. Circulation.

[53] Wu K, Wang W, Chen H, Gao W, Yu C (2019). Insulin promotes proliferation of pancreatic ductal epithelial cells by increasing expression of PLK1 through PI3K/AKT and NF-kB pathway. Biochemical and Biophysical Research Communications.

[54] Desbois-Mouthon C, Cadoret A, Blivet-Van Eggelpoël MJ, Bertrand F, Cherqui G, Perret C, Capeau J (2001). Insulin and IGF-1 stimulate the beta-catenin pathway through two signalling cascades involving GSK-3beta inhibition and Ras activation. Oncogene.

